# Pro- and Anti-Inflammatory Cytokines during Immune Stimulation: Modulation of Iron Status and Red Blood Cell Profile

**DOI:** 10.1155/2011/716301

**Published:** 2011-03-24

**Authors:** A. M. Koorts, P. F. Levay, P. J. Becker, M. Viljoen

**Affiliations:** ^1^Department of Physiology, School of Medicine, Faculty of Health Sciences, University of Pretoria, P.O. Box 2034, Pretoria 0001, South Africa; ^2^Department of Internal Medicine, Kalafong Hospital, University of Pretoria, Pretoria 0001, South Africa; ^3^Biostatistics Unit, Medical Research Council, Pretoria 0001, South Africa

## Abstract

Forty-eight patients were subdivided according to C-reactive protein (CRP) levels, resulting in 19 patients with normal (2.8 ± 2.8 mg/L) and 29 with elevated (82.2 ± 76.2 mg/L) CRP levels. The elevated CRP group had iron and red blood cell (RBC) profiles characteristic of chronic immune stimulation (CIS), and the normal CRP group, profiles of true iron deficiency. Normal relationships between storage iron, bioavailable iron, and RBC indices were absent in the elevated CRP group—implying the role of iron as major determinant of the RBC profile to be diminished during CIS. The elevated CRP group had significant increases in proinflammatory cytokines (INF-*γ*, TNF-*α*, Il-1*β*, Il-6, and Il-8). Anti-inflammatory cytokine levels were normal, except for Il-10, supporting previous indications that Il-10 contributes to reducing bioavailable iron. Regression analysis suggested decreases in transferrin to be related to increases in Il-8 and an increase in ferritin to be related to a decrease in Il-12 levels. TGF-*β* levels were positively related to transferrin and negatively to ferritin.

## 1. Introduction

Cytokine production is a feature of immune stimulation, and alterations in the cytokine profile can influence both the iron status and red blood cell profile. Chronic immune stimulation is often associated with a decrease in serum iron. Depending on the cause of the immune stimulation, the purpose of this decrease is to reduce the bioavailability of iron in order to stem uncontrolled cellular proliferation, to reduce excessive production of reactive oxygen radicals and/or to withhold iron from pathogenic microorganisms. The effects of cytokines on iron bioavailability are largely mediated through a reduction in duodenal absorption of iron and a shift in the handling of iron by the macrophage in favour of iron storage. The latter can, in time, lead to hypoferraemia [1–3] and haemosiderosis of the macrophage [2, 4, 5]. This reduction in bioavailable iron can contribute to the anaemia of chronic disease (ACD). 

It is commonly accepted that duodenal absorption of iron is decreased during chronic immune stimulation [6], and that cytokines are involved [7]. Under normal conditions, absorbed Fe^2+^ joins the intracellular labile iron pool of the enterocyte from where it can either be stored in the iron storage protein, ferritin, of the enterocyte, or translocated to the basolateral membrane for transport into the circulation [8, 9]. With chronic immune stimulation the expression of enterocyte ferritin is upregulated, resulting in excessive sequestration and entrapment of iron within the enterocyte [10]. This newly acquired enterocyte iron will subsequently be expelled from the body during sloughing of the lining of the gastrointestinal tract. In addition, the numbers of the basolateral membrane iron exporter, ferroportin, are reduced thus blocking the entry of absorbed iron into the circulation. Ferroportin expression is downregulated by the acute phase protein, hepcidin. Hepcidin release is stimulated by the cytokine interleukin-6 [11].

Iron circulates between the iron-containing compartments (intercellular iron shuttling) bound to the plasma iron transport protein transferrin. Transferrin is a negative acute phase protein, and as such serum transferrin is reduced during immune stimulation [6] resulting in less iron being available for cellular processes. Although interleukin-6 is the major cytokine responsible for the regulation of acute phase protein synthesis during chronic immune stimulation [12], other cytokines, including interleukin-1*β*, tumor necrosis factor-*α*, and interleukin-8, have also been found to moderately influence the synthesis of acute phase proteins [12–14].

Ferritin, the major intracellular protein responsible for the storage of macrophage iron, plays a major role in the establishment and maintenance of an iron transfer block in the macrophage and thus in the hypoferraemic state of chronic immune stimulation. Ferritin up-regulation precedes the reduction in serum iron [15]. The proinflammatory cytokines tumor necrosis factor-*α*, interleukin-1*β*, interleukin-6, and the anti-inflammatory cytokine interleukin-10 have all been shown to directly stimulate the transcription and translation of ferritin [16–18]. Cytokines not only upregulate ferritin expression, but also stimulate the macrophage to increase its uptake of iron by increasing the expression of the divalent metal transporter 1. Interferon-*γ* has been shown to stimulate the uptake of ferrous iron by macrophages. In addition, the anti-inflammatory cytokines, interleukin-4 and interleukin-10, can upregulate transferrin receptor expression, resulting in an increase in transferrin receptor-mediated uptake of iron by the macrophage [6, 18]. The macrophage obtains most of its iron by the degradation of haemoglobin and phagocytosis, and degradation of senescent erythrocytes is known to increase during chronic immune stimulation. This process is directly upregulated by tumor necrosis factor-*α* that stimulates the expression of C3bi (CD11b/CD18) receptors—the receptors responsible for the recognition and uptake of damaged erythrocytes. In addition, tumor necrosis factor-*α* can also indirectly upregulate this process by damaging circulating erythrocytes. These damaged erythrocytes are then phagocytosed upon binding to C3bi (CD11b/CD18) receptors [6, 18]. Not only is haemoglobin iron obtained by degradation of red blood cells, but free plasma haemoglobin is taken up by the haemoglobin scavenger receptor, CD163. Interleukin-10 and interleukin-6 augment macrophage haemoglobin acquisition by stimulating the expression of the haemoglobin scavenger receptor, CD163 [18]. In addition to increased uptake of iron by the macrophage during immune stimulation, release of iron is also reduced. Interferon-*γ* contributes to this by downregulating the expression of ferroportin, the major transmembrane protein responsible for the release of macrophage iron [18]—a process that is also affected by hepcidin [19]. Many proinflammatory mediated effects on iron homeostasis are counterbalanced by anti-inflammatory cytokines such as interleukin-4 and interleukin-13 [18].

The aim of this study was to investigate the pro- and anti-inflammatory cytokine status and the possible relationship of these cytokines to the iron status and red blood cell profiles in patients with chronic immune stimulation. A diverse group of patients with chronic disease was divided into two groups based on their C-reactive protein levels and then evaluated in terms of their iron status, red blood cell profiles, and pro- and anti-inflammatory cytokines.

## 2. Materials and Methods

### 2.1. Patients

The study group consisted of 48 patients attending the Department of Internal Medicine, Kalafong Hospital, South Africa, for treatment of chronic diseases. Blood and bone marrow were collected from each patient. The diagnosis of the patients were diverse and included various types of infections (tuberculosis (TB), malaria, human immunodeficiency virus (HIV)), cancers (lung, breast), pancytopenias as a result of bone marrow suppression or peripheral destruction of blood cells, organ failures including renal failure, heart failure and liver failure, anaemias with different etiologies, and various other pathologies. This resulted in an extremely heterogenous group of patients. The diagnosis and HIV status of all patients are presented in Table 1. Patients were subdivided based on their C-reactive protein levels. Ethical clearance for the study was obtained from the Faculty of Health Sciences Research Ethics Committee, University of Pretoria (ethical clearance number 118/2003), and all patients gave informed consent.

### 2.2. C-Reactive Protein (CRP)

CRP levels were determined by a CRP ELISA (DRG Diagnostics, Germany, Orb Diagnostics, Modderfontein, South Africa).

### 2.3. Cytokines

Interleukin-8 (Il-8), interleukin-1*β* (Il-1*β*), interleukin-6 (Il-6), interleukin-10 (Il-10), tumor necrosis factor-*α* (TNF-*α*), and interleukin-12p70 (Il-12) were determined by the Human Inflammation Kit, BD Cytometric Bead Array (CBA) (The Scientific group, Midrand, South Africa). Interleukin-2 (Il-2), interleukin-4 (Il-4), interleukin-5 (Il-5), interleukin-10 (Il-10), tumor necrosis factor-*α* (TNF-*α*), and interferon-*γ* (INF-*γ*) were determined by the BD Cytometric Human T-helper cell type-1/T-helper cell type-2 Cytokine Kit (The Scientific group, Midrand, South Africa). With the T-helper cell type-1/T-helper cell type-2 CBA cytokine kit, the interferon-*γ* standards were lost and the measurement of INF-*γ* was done by the human INF-*γ* ELISA Kit, BD OptEIA test (The Scientific group, Midrand, South Africa). Il-10 and TNF-*α* were measured in both the Human Inflammation kit and the Human T-helper cell type-1/T-helper cell type-2 kit. The mean of these values were calculated and used for statistical analysis. Transforming growth factor-*β*1 (TGF-*β*1) was determined by a TGF-*β*1 ELISA (DRG Diagnostics, Germany, Orb Diagnostics, Modderfontein, South Africa). Granulocyte macrophage-colony stimulating factor (GM-CSF) was determined by a GM-CSF ELISA (DRG Diagnostics, Germany, Orb Diagnostics, Modderfontein, South Africa).

### 2.4. Serum Iron Markers

Serum iron markers were determined by the Chemical Pathology laboratory, National Health Laboratory Services (NHLS), University of Pretoria, South Africa.

### 2.5. Soluble Transferrin Receptor

The soluble transferrin receptor was determined by an ELISA from Ramco Laboratories, Inc., Texas, USA.

### 2.6. Prussian Blue Iron Stains of Bone Marrow Aspirates and Core Bone Marrow Biopsies

#### 2.6.1. HCl-Ferrocyanide Iron Stains of Bone Marrow Aspirate Smears

The iron stains and evaluation of the bone marrow aspirate smears were performed by the Haematology laboratory, NHLS, University of Pretoria, South Africa.

#### 2.6.2. HCl-Ferrocyanide Iron Stains of Core Bone Marrow LR White Plastic Sections

A piece of core bone marrow was obtained during the time the patients had their biopsies, taken for diagnostic purposes, and placed immediately in the fixative on ice. The fixative consisted of 4% formaldehyde (FA) and 0.05% glutaraldehyde (GA) in a 0.15 M sodium phosphate buffer. The fixative was prepared fresh immediately prior to the obtainment of bone marrow tissue. A 10% paraformaldehyde solution in deionised H_2_O was prepared fresh in a fume hood (Paraformaldehyde (Trioxymethylene), SPI Supplies, cat. no. 2615, Rick Loveland & Associates, Halfway House, South Africa). The solution was heated to 60–70°C with constant stirring. Once the solution had reached the proper temperature stirring was continued for 15 minutes. At this point, the solution was milky. One to two drops of 1 N NaOH was added, with stirring, until the solution cleared [20]. The 0.15 M sodium phosphate buffer was prepared from two stock solutions, a 0.3 M Na_2_HPO_4_ stock solution (di-Sodium hydrogen phosphate Dihydrate, Fluka, BioChemika, Ultra, cat. no. 71643, Sigma-Aldrich, Aston Manor, South Africa) and a 0.3 M NaH_2_PO_4_ stock solution (Sodium dihydrogen phosphate Dihydrate, Fluka, Biochemika, MicroSelect, cat. no. 71505, Sigma-Aldrich, Aston Manor, South Africa). The 0.3 M NaH_2_PO_4_ stock solution was added to the 0.3 M Na_2_HPO_4_ stock solution to a pH of 7.25 immediately prior to the obtainment of bone marrow tissue. This 0.3 M sodium phosphate buffer solution was then diluted 1 : 1 with the 10% freshly prepared formaldehyde stock solution and deionised H_2_O. This was followed by the addition of the GA (Pure Glutaraldehyde 25% solution, E.M. grade, SPI Supplies, cat. no. 2607, Rick Loveland & Associates, Halfway House, South Africa). The bone marrow tissue was fixed for 24 hours at 6°C in this fixative. All subsequent steps were carried out at 6°C with rotation (TAAB rotator, Wirsam Scientific, Richmond, South Africa). After 24 hours, the bone marrow tissue was washed 3 times for 20 minutes with the sodium phosphate buffer. It was then dehydrated as follows: 50% EtOH, 70% EtOH, 30 minutes each, followed by 85% EtOH, twice for 15 minutes each (Ethanol 99.9% Absolute A.R., Minema, Rick Loveland & Associates, Halfway House, South Africa). Subsequently, the bone marrow tissue was infiltrated with a 1 : 1 85% EtOH : LR White mixture for 30 minutes. LR White dissolved in 85% EtOH but not in 80% EtOH (LR White Resin, medium grade acrylic resin, London Resin Company LTD., Rick Loveland & Associates, Halfway House, South Africa). The bone marrow tissue was infiltrated with LR White, twice for 30 minutes each. The tissue was then placed in gelatine capsules in fresh LR White ensuring no air bubbles and polymerised for 24 hours at 50°C (Gelatine capsules, SPI Supplies, cat. no. 2302, Rick Loveland & Associates, Halfway House, South Africa). The block of bone marrow tissue was sectioned into 2 *μ*m thick sections which were then placed on microscope slides (Menzel-Glaser Superfrost Plus Microscope Slides, Labotec, Halfway House, South Africa). The slides were rinsed in deionised H_2_O before the staining procedure. The sections were stained for 1 hour in 10% ferrocyanide/10% HCl prepared just before use in Coplin jars at 25°C (10% ferrocyanide, Potassium hexacyanoferrate (II) Trihydrate, Fluka, Biochemika Ultra, cat. no. 60279, Sigma-Aldrich, Aston Manor, South Africa; 10% HCl, Hydrochloric acid 30%, Riedel-de-Haën, cat. no. 30053, Sigma-Aldrich, Aston Manor, South Africa). After the staining step, the slides were rinsed in deionised H_2_O. The sections were counterstained with 1% eosin in 70% ethanol (acidified) for 10 minutes (1% eosin, Eosin yellowish, Gurr, Microscopy Material, cat. no. 45380, BDH Chemicals Ltd., England, in 70% ethanol, acidified with acetic acid). Once again, the slides were rinsed in deionised H_2_O. Finally, the slides were air-dried on a hot plate and mounted with immersion oil and a cover slide.

### 2.7. Red Blood Cell Profile

Red blood cell counts, haemoglobin concentration, mean corpuscular volume (MCV), mean corpuscular haemoglobin (MCH), mean corpuscular haemoglobin concentration (MCHC), and red blood cell distribution width (RDW) were determined by the NHLS, University of Pretoria, South Africa.

### 2.8. Statistical Analysis

The ranksum test was employed on log transformed data. This was done due to the skewness of the data sets. For summary statistics, the geometric mean and the 95% confidence interval were reported. Pearson's product-moment correlation coefficient (*r*) was employed to assess dependence between study parameters. Testing was done at the 0.05 level of significance. Forward stepwise regression with *pe* = 0.2 as the entrance point was employed to investigate the relationship between each of the iron status and red blood cell profile parameters and the pro- and anti-inflammatory cytokines. *pe* specifies the significance level for addition to the model; thus, terms with *P* < .2 are eligible for addition. The discrepancy between *R* ^2^ and adjusted *R* ^2^ was due to the small sample size.

## 3. Results

The patients were subdivided according to the immunological indicator C-reactive protein into a group with normal (≤10 mg/L) and a group with elevated (>10 mg/L) C-reactive protein levels. This resulted in a group of 19 patients with normal C-reactive protein (2.8 ± 2.8 mg/L) and a group of 29 patients with elevated C-reactive protein (82.2 ± 76.2 mg/L). The average and SD for age for the group of patients with elevated C-reactive protein was 41.6 ± 15.9 years and for the group of patients with normal C-reactive protein 42.3 ± 15.2 years. The majority of patients were black females, with the exception of four males and three whites and six males and one white in the elevated C-reactive protein and normal C-reactive protein groups, respectively.

The results for the pro- and anti-inflammatory cytokines are presented in Table 2. Various proinflammatory cytokines including INF-*γ*, TNF-*α*, Il-1*β*, Il-6, and Il-8 were elevated in the group of patients with elevated C-reactive protein compared to the group of patients with normal C-reactive protein. No significant differences were shown for the proinflammatory cytokines Il-2 and Il-12. The T-helper cell type-2 cytokines involved in predominantly anti-inflammatory processes including Il-4, Il-5 and TGF-*β*, were not significantly different between the group of patients with elevated C-reactive protein and the group of patients with normal C-reactive protein. However, the T-helper cell type-2 cytokine, Il-10, was significantly higher in the group of patients with elevated C-reactive protein compared to the group of patients with normal C-reactive protein.

The iron status of the group of patients with elevated C-reactive protein was characteristic of patients with a chronic proinflammatory immune status. The serum iron markers and soluble transferrin receptor for the two groups of patients are shown in Table 3. Serum transferrin levels were significantly lower, serum ferritin significantly higher, and soluble transferrin receptor significantly lower in the group with elevated C-reactive protein.

When the red blood cell profiles of the group with normal C-reactive protein and the group with elevated C-reactive protein were compared (Table 4), it was seen that both groups were anaemic, but that the MCV, MCH, and the MCHC were significantly lower in the group of patients with normal C-reactive protein. The RDW did not differ significantly between the groups, but was higher than normal for both groups.

To assess total body iron stores, in order to distinguish between true iron deficiency and an iron transfer block, a Prussian blue iron stain was performed on the bone marrow aspirates and bone marrow cores. Results can be seen in Table 5.

Various correlations were shown between storage iron, bioavailable iron, and red blood cell production in the group of patients with normal C-reactive protein. None of these correlations could be demonstrated in the group of patients with elevated C-reactive protein (refer to Table 6).

The dependence of various serum iron markers, soluble transferrin receptor, and red blood cell indices on pro- and anti-inflammatory cytokines in the group of patients with elevated C-reactive protein are presented in Table 7. 

## 4. Discussion

C-reactive protein is an acute phase protein and rises sharply with the onset of inflammation reaching peak concentrations within 24–48 hours. Inflammatory processes accompanying tissue injury, infection, malignancy, autoimmune diseases, and cardiovascular diseases can all result in an increase in C-reactive protein levels [21]. C-reactive protein is synthesised and secreted by hepatocytes upon stimulation by acute phase protein-inducing cytokines such as Il-6 [22]. In patients with elevated C-reactive protein, the macrophage takes on a proinflammatory role, which is characteristic of a T-helper cell type-1 immune response. An increase in C-reactive protein is thus seen as an indicator of the involvement of the classically activated macrophage (proinflammatory macrophage). C-reactive protein levels are considered by some as the most accurate reflection of the inflammatory state [23].

In the present study, patients with chronic disease were divided into two groups based on their C-reactive protein levels and then evaluated in terms of their iron status, red blood cell profiles, and pro- and anti-inflammatory cytokines.

Iron status was evaluated by Prussian blue iron stains of bone marrow aspirates and cores, by levels of serum iron markers, and by red blood cell indices. The results are shown in Tables 3, 4, and 5. In the group of patients with high C-reactive protein levels, a high prevalence of iron transfer block was found when all factors were considered. The prevalence was 69% for the patients with elevated C-reactive protein, in contrast to the 26% for the group with normal C-reactive protein levels. The majority of the patients with high C-reactive protein levels had serum iron profiles characteristic of patients with an iron transfer block [6], that is, a decrease in serum iron, an increase in ferritin, a decrease in transferrin, a small increase in soluble transferrin receptor, a decrease in transferrin/log ferritin ratio, and a decrease in soluble transferrin receptor/log ferritin ratio. As was expected, most of the patients with normal C-reactive protein did not have serum iron profiles characteristic of an iron transfer block. However, these patients had a high incidence of true iron deficiency. For these patients, a decreased serum iron, normal ferritin, normal transferrin, a marked increase in soluble transferrin receptor, an increase in transferrin/log ferritin ratio, and an increase in the soluble transferrin receptor/log ferritin ratio were shown.

Chronic immune stimulation has a negative effect on red blood cell production. Immune-stimulated decreases in bioavailable iron contribute, but other factors, mostly cytokine-induced, are also involved. Such factors include suppression of the proliferation of erythroid progenitor cells, a decrease in the synthesis of erythropoietin, a decrease in the sensitivity of erythroblasts to erythropoietin, and shortened red blood cell life span [24–26]. The anaemia of chronic disease is, therefore, on the one hand, the result of a decrease in iron reaching the erythron and, on the other, that of a suppression of red blood cell synthesis [24–26].

In this study, anaemia was present in the majority of patients, irrespective of their C-reactive protein levels. In the patients with normal C-reactive protein the inclusion of a couple of patients with different red blood cell pathologies, such as macrocytic anaemia, resulted in a normal mean corpuscular volume for the group. However, both the mean corpuscular haemoglobin and mean corpuscular haemoglobin concentration were decreased, which is characteristic of the anaemia of true iron deficiency. In the patients with elevated C-reactive protein, the average for the mean corpuscular volume corresponded to that of a normocytic red blood cell profile. It is known that patients with the anaemia of chronic disease (ACD) often exhibit a normocytic, normochromic anaemia when they are initially seen in medical care facilities [6, 27]. With further development of ACD, these patients develop a microcytic, hypochromic anaemia. In view of the iron status of the two groups, it would be reasonable to assume that the group with normal C-reactive protein had true iron deficiency anaemia, while the anaemia of the high C-reactive protein group was predominantly that of chronic disease. Il-1*β*, TNF-*α*, and TGF-*β* have been shown to inhibit erythropoietin synthesis and action [28]. In the present study, there were, as will be discussed later, increases in Il-1*β* and TNF-*α* levels in the group of patients with high C-reactive protein levels.

An important difference with regard to the relationship between iron availability and the red blood cell profile was observed between the group with elevated C-reactive protein levels and that with normal levels. The normal expected relationships were found in the group with normal C-reactive protein levels. For instance, the correlations between transferrin saturation and MCV (0.62, 0.005), transferrin saturation and MCH (0.64, 0.003), and transferrin saturation and MCHC (0.49, 0.033) supported the notion that an increase in the availability of iron has a positive effect on the red blood cell profile [29–31]. The negative correlations seen between serum transferrin and MCV (−0.62, 0.005), serum transferrin and MCH (−0.74, 0.0003), and serum transferrin and MCHC (−0.79, 0.001) reflected the fact that an increase in serum transferrin, related to iron-deficient iron stores, generally results in iron-deficient erythropoiesis [29–31]. In addition, the following negative correlations, between soluble transferrin receptor and MCV (−0.65, 0.002), soluble transferrin receptor and MCH (−0.75, 0.0002), and soluble transferrin receptor and MCHC (−0.75, 0.0002) demonstrated the increase in soluble transferrin receptor levels normally found with deficient red blood cell production [31]. These normal relationships did not exist in the high C-reactive protein group (Table 6). The results of this study thus confirm bioavailability of iron to be a major determinant of the red blood cell profile. However, with pronounced proinflammatory activity (significantly elevated C-reactive protein), accompanied by an iron transfer block and ACD, this dominancy of bioavailable iron in the regulation of proper red blood cell production was lost.

In this study, results on the cytokine profiles supported the subdivision into a normal and a proinflammatory group on the basis of C-reactive protein levels. Most of the proinflammatory cytokines, including INF-*γ*, TNF-*α*, Il-1*β*, Il-6, and Il-8, were significantly higher in the group of patients with elevated C-reactive protein compared to the group of patients with normal C-reactive protein. These cytokines are known to play a role in the reduction of available iron during chronic immune stimulation. INF-*γ* has, for instance, been shown to downregulate the expression of the transmembrane protein ferroportin [18]. Il-1*β*, TNF-*α*, and Il-6 have been reported to directly stimulate the transcription and translation of ferritin [16, 17], while Il-1*β*, TNF-*α*, Il-6, and Il-8 stimulation are known to contribute to the synthesis of acute phase proteins [12–14]. Furthermore, TNF-*α*, has been shown to increase the acquisition of erythrocyte iron by the macrophage by increasing the expression of C3bi (CD11b/CD18) receptors [6, 18]. Il-6, in addition to its role as major cytokine responsible for the regulation of acute phase protein synthesis [12], also stimulates the release of hepcidin [11], as well as the expression of the haemoglobin scavenger receptor, CD163 [18]. The proinflammatory cytokine results of this study are, therefore, in line with previous findings.

The only proinflammatory cytokines that were not significantly higher in the group of patients with elevated C-reactive protein were Il-2 and Il-12. This may perhaps be explained by the inclusion of many HIV-positive patients (15 of 29 patients) in this group. With human immunodeficiency virus infection, the production of both Il-2 and Il-12 are reduced [32, 33].

The T-helper cell type-2 cytokines, Il-4, Il-5 and TGF-*β*, were not significantly different between the patients with elevated C-reactive protein and the patients with normal C-reactive protein. However, Il-10, was significantly higher in the group with elevated C-reactive protein. This finding is in agreement with previous indications that the anti-inflammatory cytokine interleukin-10 can contribute to the hypoferraemia of chronic immune stimulation by up-regulating the transcription and translation of ferritin and by stimulating the expression of the haemoglobin scavenger receptor, CD163 [6, 18].

The above results supported the notion that T-helper cell type-1/proinflammatory cytokines are the major role players in the development of an iron transfer block. However, indications such as the raised levels of Il-10 and the observation that the iron transfer block was also seen in a number of patients with normal C-reactive protein levels, support previous suggestions [6, 18] that T-helper cell type-2 cytokines also play a role in the development/maintenance of an iron transfer block.

Regression analysis showed both serum transferrin and serum ferritin to be highly associated with the levels of a number of cytokines. For the proinflammatory cytokine, Il-8, there was a significant association with serum transferrin, with an increase in Il-8 related to a reduction in transferrin. This is in line with the assumed roles of the proinflammatory cytokines in bringing about a decrease in serum transferrin during chronic immune responses. For the proinflammatory cytokine, Il-12, there was a significant association with serum ferritin, with an increase in ferritin related to a decrease in Il-12. This might be explained by the possible immunosuppressive role of ferritin in HIV/AIDS [34]. In the present study, no significant difference were found for TGF-*β* levels between the patients with elevated C-reactive protein and patients with normal C-reactive protein. However, in the patients with elevated C-reactive protein it was shown that both transferrin and ferritin levels were related to TGF-*β* levels. TGF-*β* levels were positively related to transferrin levels and negatively to ferritin levels. This may perhaps suggest TGF-*β* to play a role in control of the proinflammatory response.

## 5. Conclusions

Patients with elevated C-reactive protein levels have a predominantly proinflammatory cytokine profile. C-reactive protein levels are thus a good general reflection of proinflammatory status. The majority, but not all, of patients with high C-reactive protein have an iron transfer block. The results support previous indications that, during chronic immune stimulation, the anti-inflammatory cytokine, Il-10, contributes to reducing bioavailable iron. The proinflammatory cytokine Il-8 might be involved in decreasing of transferrin. Ferritin, as an immunosuppressive agent, may play a role in the decrease of Il-12 production. TGF-*β* appears to play a role in the control of the proinflammatory response. During overt proinflammatory conditions the normal relationships between storage iron, bioavailable iron, and red blood cell indices are disrupted and iron bioavailability may lose its role as major determinant of the red blood cell profile. The latter finding has implications for the administration of iron to patients with ACD.

## Figures and Tables

**Table 1:** Diagnosis and HIV status of the patients (*elevated CRP).

Patient	Diagnosis	HIV
1*	Pneumonia and sepsis, Escherichia coli and urinary tract infection and acute renal failure	pos
2*	Heart failure and megaloblastic anaemia and pernicious	neg
3*	Anaemia and idiopathic thrombocytic purpura and acute haemolytic anaemia versus disseminated intravascular coagulopathy, post mortem	pos
4*	Carcinoid cancer and pneumonia and metastasis to the brain	neg
5*	Idiopathic vasculitis and pancytopenia questioning antiphospholipid syndrome and uterine mass	neg
6	Malaria and idiopathic thrombocytic purpura	neg
7*	Cruveilhier-Baumgarten disease and hypersplenism and TB	neg
8*	Lung cancer and acute renal failure and pneumonia	neg
9*	Pneumonia, Escherichia coli	pos
10	Idiopathic thrombocytic purpura and iron deficient anaemia and questioning systemic lupus erythematosus	neg
11*	Retroviral disease and renal failure	pos
12	Metastatic breast cancer	neg
13*	Pneumonia and confusion	pos
14*	Pulmonary TB and effusion	pos
15	Megaloblastic anaemia	neg
16	Anaemia due to blood loss	neg
17	Retroviral disease and anaemia and Kaposi's sarcoma and previous pulmonary TB	pos
18*	Antiphospholipid syndrome and haemolytic anaemia	neg
19*	Retroviral disease and bicytopenia and mycobacterium avium complex	pos
20*	Retroviral disease and anaemia and dilated cardiomyopathy and antiphospholipid syndrome and TB and thrombosis	pos
21*	Retroviral disease and TB and sepsis and anaemia and ascitis	pos
22*	Chronic obstructive pulmonary disease and liver and kidney failure and pelagra and sepsis and ethanol abuse	neg
23*	Retroviral disease and pneumonia and pancytopenia	pos
24	Idiopathic thrombocytic purpura	neg
25	Retroviral disease and diabetes mellitus and heart failure and obesity and splenomegaly and lymphadenopathy and TB, bone marrow	pos
26*	Ethanol abuse and radial fracture and pulmonary TB and bradycardia and primary hypertension, increase calcium	neg
27	Massive splenomegaly and pancytopenia	neg
28*	Megaloblastic anaemia and syphilis	neg
29*	Delerium and TB and calcified nodes, post mortem = miliary TB	neg
30*	OD and thrombocytopenia and macrocytosis and ethanol abuse	neg
31*	Retroviral disease and pulmonary TB and pancytopenia	pos
32	Nephritis: hypertension, edema, proteinuria	neg
33*	Pancytopenia and ascitis and TB and proteinuria and urinary tract infection and sepsis, ICU	neg
34*	Retroviral disease and Hodgkin's disease and TB	pos
35	Hypertension and diabetes mellitus and leucocytosis, persistent	neg
36*	Retroviral disease on HAART and pulmonary TB	pos
37	Retroviral disease and idiopathic thrombocytic purpura-immune, normal spleen	pos
38	Megaloblastic anaemia and hypothyroidism	neg
39	Anaemia	neg
40*	Anaemia and pyrexia and miliary TB, bone marrow culture	pos
41	Pancytopenia and idiopathic thrombocytic purpura	pos
42	Iron-deficient anaemia	neg
43	Iron-deficient anaemia and peptic ulcer disease and pneumonia, Staphylococcus aureus	neg
44*	Idiopathic 4-limb African gangrene	neg
45	Retroviral disease and pneumonia and pancytopenia	pos
46*	Monoclonal gammopathy of undetermined significance and uterine mass	neg
47*	Retroviral disease and pancytopenia and pneumonia	pos
48	Idiopathic thrombocytic purpura and bicytopenia	neg

**Table 2:** Cytokine levels for patients with elevated C-reactive protein and patients with normal C-reactive protein.

	Elevated CRP (*n* = 28) Geometric mean; 95% confidence interval	Normal CRP (*n* = 19) Geometric mean; 95% confidence interval	*P*-value
INF-*γ*	2.00; 0.76–5.27	0.18; 0.09–0.37	.001
TNF-*α*	3.47; 2.78–4.32	2.29; 1.88–2.78	.009
Il-1*β*	1.81; 0.92–3.57	0.49; 0.26–0.92	.008
Il-6	79.23; 36.69–171.09	3.56; 2.08–6.09	.000
Il-12	2.69; 1.69–4.28	2.73; 1.50–4.99	.97
Il-2	7.41; 4.85–11.33	5.04; 2.45–10.37	.31
Il-8	88.12; 49.84–155.80	14.20; 8.80–22.91	.000
GM-CSF	2.15; 1.21–3.81	2.58; 0.89–7.44	.73
Il-4	1.74; 1.21–2.51	1.48; 0.87–2.50	.59
Il-5	2.35; 1.74–3.16	2.23; 1.18–4.19	.86
TGF-*β*	10.51; 8.34–13.24	8.60; 6.34–11.67	.28
Il-10	9.37; 5.66–15.51	4.56; 3.29–6.32	.034

**Table 3:** Serum iron markers and soluble transferrin receptor for patients with elevated C-reactive protein and patients with normal C-reactive protein.

Normal laboratory values	Elevated CRP (*n* = 28) Geometric mean; 95% confidence interval	Normal CRP (*n* = 19) Geometric mean; 95% confidence interval	*P*-value
Serum iron (10–30 *μ*mol/L)	6.87; 5.11–9.22	7.89; 5.15–12.08	.57
Serum transferrin (2–3.6 g/L)	1.21; 1.04–1.41	2.33; 1.98–2.75	.000
Transferrin saturation (15–50% f, 20–50% m)	21.59; 14.90–31.26	14.34; 8.48–24.25	.18
Serum ferritin (11–306.8 *μ*g/L f, 23.9–336.2 *μ*g/L m)	928.70; 482.12–1788.95	44.97; 19.31–104.71	.000
Transferrin/log ferritin	0.43; 0.34–0.54	1.59; 1.07–2.38	.000
Soluble transferrin receptor (2.9–8.3 *μ*g/mL)	7.41; 5.69–9.67	13.38; 9.30–19.24	.008
Soluble transferrin receptor/log ferritin	2.58; 1.94–3.43	9.14; 5.22–16.00	.000

**Table 4:** Red blood cell indices for patients with elevated C-reactive protein and patients with normal C-reactive protein.

Normal laboratory values	Elevated CRP (*n* = 28) Geometric mean; 95% confidence interval	Normal CRP (*n* = 19) Geometric mean; 95% confidence interval	*P*-value
Red blood cell count (4.13–5.67 × 10^12^/L f, 4.89–6.11 × 10^12^/L m)	2.33; 1.95–2.78	2.62; 2.04–3.36	.42
Haemoglobin (12.1–16.3 g/dL f, 14.3–18.3 g/dL m)	6.80; 5.66–8.16	6.23; 4.74–8.19	.57
Haematocrit (0.370–0.490 L/L f, 0.430–0.550 L/L m)	0.21; 0.18–0.25	0.21; 0.16–0.27	.94
Mean corpuscular volume (79.1–98.9 fL)	90.05; 84.42–96.05	78.98; 72.01–86.62	.016
Mean corpuscular haemoglobin (27–32 pg)	29.24; 27.09–31.55	23.82; 21.13–26.84	.003
Mean corpuscular haemoglobin concentration (32–36 g/dL)	32.47; 31.70–33.26	30.16; 29.00–31.38	.001
Red blood cell distribution width (11.6–14%)	19.39; 17.48–21.51	21.34; 18.38–24.77	.27

**Table 5:** Iron stores and prevalence of iron transfer block for patients with elevated C-reactive protein and patients with normal C-reactive protein.

	Elevated C-reactive protein *n* = 29	Normal C-reactive protein *n* = 19
Increased iron stores	59%	16%
Normal iron stores	22%	21%
Decreased iron stores	19%	63%
Iron transfer block	69%	26%
No iron transfer block	31%	74%

**Table 6:** Correlations between storage iron, bioavailable iron, and red blood cell production in patients with normal C-reactive protein levels and patients with elevated C-reactive protein.

	Serum iron		Serum transferrin		Mean corpuscular volume		Mean corpuscular haemoglobin		Mean corpuscular haemoglobin concentration	
	*r*-value; *P*-value		*r*-value; *P*-value		*r*-value; *P*-value		*r*-value; *P*-value		*r*-value; *P*-value	
	N CRP	↑ CRP	N CRP	↑ CRP	N CRP	↑ CRP	N CRP	↑ CRP	N CRP	↑ CRP
Serum transferrin					−0.62; .005	−0.03; .88	−0.74; .0003	−0.08; .68	−0.79; .0001	−0.17; .39
Transferrin saturation					0.62; .005	0.43; .030	0.64; .003	0.48; .013	0.49; .033	0.33; .10
Transferrin/log ferritin					−0.66; .002	0.08; .69	−0.72; .0005	0.03; .88	−0.70; .001	−0.11; .57
Soluble transferrin receptor	−0.52; .024	0.14; .47	0.59; .007	0.11; .59	−0.65; .002	−0.04; .85	−0.75; .0002	−0.08; .69	−0.75; .0002	−0.18; .35
Soluble transferrin receptor/log ferritin					−0.60; .007	0.06; .77	−0.65; .003	−0.003; .99	−0.61; .005	−0.18; .35

**Table 7:** Forward stepwise regression for serum iron markers and red blood cell indices in terms of pro- and anti-inflammatory cytokines (*significant, ^*√*^non-significant, and coefficient values).

	INF-*γ*	TNF-*α*	Il-1*β*	Il-6	Il-12	Il-2	Il-8	GM-CSF	Il-4	Il-5	TGF- *β*	Il-10	**R^2^**	**Adjusted R^2^**
Serum iron													0.000	0.000

			*√*				∗				∗		0.51	0.45
Serum transferrin			0.05				-0.09				0.39			

											∗		0.17	0.13
Transferrin saturation											-0.61			

	*√*			*√*	∗		*√*		*√*	*√*	∗	*√*	0.73	0.62
Serum ferritin	0.16			-0.24	-0.46		0.32		0.58	0.45	-0.81	0.33		

		*√*			∗		*√*	*√*			∗		0.61	0.52
Transferrin/log ferritin		-0.38			0.21		-0.12	0.09			0.38			

Soluble transferrin	*√*						*√*	*√*					0.25	0.16
receptor	0.11						-0.12	0.13						

Soluble transferrin		∗					*√*			∗	*√*		0.39	0.28
receptor/log ferritin		0.50					-0.13			-0.37	0.31			

											∗	∗	0.28	0.23
Red blood cell count											0.28	-0.13		

											*√*	∗	0.29	0.23
Haemoglobin											0.19	-0.17		

			*√*								*√*	∗	0.36	0.28
Haematocrit			-0.06								0.22	-0.15		

									∗				0.22	0.19
Mean corpuscular volume									-0.08					

Mean corpuscular									∗	*√*	*√*		0.28	0.19
haemoglobin									-0.08	-0.06	-0.10			

Mean corpuscular					*√*						∗	*√*	0.33	0.24
haemoglobin concentration					0.02						-0.04	-0.01		

Red blood cell								*√*				∗	0.29	0.23
distribution width								0.05				0.10		
